# Meat Inspection1A paper presented to the New York State Veterinary Medical Society at Albany, N. Y., September 13, 1895.

**Published:** 1895-02

**Authors:** Claude D. Morris


### MEAT INSPECTION.[1]

BY CLAUDE D. MORRIS, V.S.

Mr. President and Gentlemen: The question of meat inspection, discussed from a sanitary standpoint, is one of paramount importance in the annals of social economy. The capabilities of a man to exercise his mental and corporal functions normally depend, to a large degree, upon the wholesomeness of the food upon which he daily subsists. Without argument, if this is true, it becomes none the less a fact when applied to the Commonwealth. Therefore, six and one-half millions of people fed on wholesome, life-giving food become strong in body and mind, energetic, and contented, not likely to be given to riots, strikes, and lawlessness; but, on the contrary, pursue a life of honest toil, frugality, and happiness. Visit the slums of our cities and those who dwell therein, examine their dietary, and you will conclude at once that this is a fitting place as the origin of Coxeyites, lawless mobs, and discontent. One of the questions endangering our national life to-day, which is demanding adjustment in the halls of legislation, is the evil arising from cheap labor. Cheap labor means cheap food always, and what we mean by cheap food is an article that is not standard, and is unwholesome to a greater or less degree. We sincerely believe that it is not a far-fetched argument that within the elements of inferior food lurk the seeds of socialism. It was our privilege, during the excitement of the recent strike, to overhear a conversation by a band of workmen employed on the public works in the vicinity of the sub-treasury, Wall Street, New York City. As they sat on the curb at noon, eating their lunch, composed of cheap "Bologna" and black bread, one of the number remarked that instead of their being there with a pick and shovel under those depressing influences, referring to the nature of his noon meal and the heat of the day, making roads over which the commerce of that great city rolls, they should have in their hands implements of war, instead of industry, to destroy that sub-treasury and divide among themselves its contents, the yellow metal. We venture to assert that had these men been

[1] A paper presented to the New York State Veterinary Medical Society at Albany, N. Y., September 13, 1895.

sitting at a table eating a wholesome piece of meat, instead of cheap "Bologna," even with black bread, they would have been engaged in conversation the converse of such dire and hellish designs, such as are born only of socialism.

One of the acknowledged and defined laws of Nature governing the physical activities of human existence precludes from the beginning the over-taxing of the physical nature, resulting in a corresponding attenuation of the mental functions. This, then, being one of the infallible laws of our being, we are furnished with a solution of the problem that the constantly employed physical laborer is not the thinking man of a community, the over-taxing of his bodily energies having resulted in a lessening of the mental functions. In this condition, then, if there is any one thing which will arouse a spirit of envious discontent and a belief that laws are unjust and oppressive, that the distribution of wealth and luxury is unequal, it is when an over-taxed body, accompanied with a weak mind, is eating a spare meal of inferior food. That man is in no condition to reason out the wisdom of the Scriptural injunction "For he that hath, to him shall be given, and he that hath not, from him shall be taken even that which he hath." I shall not reason longer along this line; sufficient only to convey the idea that we believe if the laboring and poorer classes were fed on wholesome food the germs of socialism would not find a domicile in our land.

Our subject is one of the questions which make up the integral of sanitation. Sanitary laws demand recognition in every phase of our existence. We find it is one of the oldest of sciences. The hygienic laws handed down by Moses and Hippocrates are to-day just as applicable as they were two thousand years ago, and only need adaptation to our complex civilization. Nevertheless, progress in the practical application of sanitary principles has not been rapid. It has kept pace with the advance of knowledge; but this has of necessity been slow and disappointing. Until the newspapers began to be widely diffused there was no means of popular enlightenment, and even now people are apathetic about hygienic matters and neglect the "ounce of prevention" nowhere else so vitally important, and especially so in its relation to the question of meat inspection, the gastronomic taste of our Commonwealth is quite animal, and on no question affecting our social economy is the public more ignorant. On that ground alone it is encumbent upon our sanitary authorities to protect the people against fraud and disease. As we have stated, and it is an acknowledged physiological fact, that a diet so largely albuminous as that of animal flesh must of necessity become, to a large extent, an indirect civilizing force. Wholesome meats are of the greatest importance in the maintenance of health; while, on the other hand, diseased meats, without doubt, environ society with many dangers.

It is difficult for the general public to obtain sufficient knowledge, as a means of self-protection, while busily engaged in the diversified activities of life so fraught with labor and responsibilities as that of our American citizenship. In the husbandry of our Commonwealth the purchaser with his daily wages in his pocket and the cravings of hunger gnawing at his stomach is not in a position to discriminate in a manner always judicious in the purchase of the necessaries of life, nor could he expect to pay for expert services to determine the wholesomeness or otherwise of the food he is about to select, which is to satisfy the natural appetite of himself and family; but, on the other hand, is most likely to purchase that which, to his uneducated sense, is to supplant those daily longings of which he and his family are cognizant—hunger—trusting largely to the integrity of the butcher as to the fitness of animal flesh as food. But we find there are numerous conditions which are patent to the most casual observer already existing in the meat trade, especially so in our large cities, which now demand adjustment, conditions which should no longer give place to the credulity of innocent consumers. We conclude, therefore, that meat inspection is of necessity, and it must necessarily enter the arena of sanitary science. It is necessary, also, because of the prevalence of disease among animals whose flesh is used as human food—diseases that are contagious and infectious among animals themselves and communicable to man. In the next place, what are the diseases the animal economy is prone to that scientific investigation has proven as a menace to human health, and what should be condemned as unfit for human food, and what should be considered as wholesome? In endeavoring to answer these questions we realize at the outset that there is a difference of opinion regarding certain diseases and their toxic effect in meat; but it is our aim to consider only those diseased conditions which, when brought in contact or through alimentation with man, have been proven, and are so accepted to be, nocuous as food.

As a question to be answered as a whole, the one already stated becomes the most difficult of all we have to deal with in connection with the inspection of meat. While we may possess all the proof indirectly of the unwholesomeness of certain flesh, yet we may lack one link in the chain of evidence in supporting the fact of its noxiousness. On an occasion like this we shall generalize the proof in support of our position. We take it for granted that all will admit, where neat cattle having been slaughtered while suffering from inflammatory or febrile diseases, also important forms of blood deterioration or degradation must, of necessity, based either on sentiment or science, admit of its unfitness for human food. The diseases to which we shall allude are pneumonia, simple, sporadic, and contagious, including other lung diseases, suppurative diseases of the tissue or organs of the body. A well-defined cachexia, whether it be cancerous or tuberculous, also where there is advanced putrefaction or mortification of tissue or parts of organs; septicæmia, pyæmia, erysipelas, anthrax, variola, uræmia, dropsy, hydatid disease of the muscles, parturient apoplexy, cattle plague, actinomycosis, foot and mouth diseases, hog cholera, and swine plague. We should recognize that although meat may not be particularly nocuous, it may be quite as harmful in an indirect way by being innutritious. Perhaps some may doubt the advisability of condemning the flesh of neat cattle though suffering with pulmonary and uræmic diseases; but when we come to consider the function of the lungs and kidneys in health and the relation they bear in the maintenance of animal vigor, it seems to us that we should as unhesitatingly condemn as nocuous the same as the more toxic diseases, such as pyæmia, septicæmia, and erysipelas, or puerperal and carbuncular fevers of the anthracoid type. For myself, I believe that whenever flesh is offered for sale as human food, having been slaughtered while suffering from diseases and under medical treatment, it should be condemned on general principles, because the vast majority of neat cattle offered for sale as food while under such conditions are prognosed as unfavorable, and it is the last resort of the owner to realize something as an indemnity, knowing that it will not reach his table, and caring less for the injustice he has imposed upon the innocent consumer. The very existence of such a market cultivates this vicious desire on the part of some cattle dealers and sears all sense of justice as to its evil effect in the community. While such argument may seem to be based on sentiment, it does possess its grain of justice. On the other hand, for sanitary reasons, we must all admit that such meats are quite liable to produce all sorts of enteric disturbances and fevers.

The Secretary of Agriculture on September 13, 1893, issued instructions to inspectors of the Bureau of Animal Industry in charge of the inspection of animals slaughtered at abattoirs throughout the United States, a list of diseases and other conditions as a basis for condemnation, either on ante- or post-mortem examinations. The last clause of these instructions reads as follows: "12. Any disease or injury causing elevation of temperature or affecting the system of the animal to a degree which would make the flesh unfit for human food, any organ or part of a carcass which is badly bruised or affected by tuberculosis, actinomycosis, abscess, suppurating sore, or tape-worm cysts, should be condemned." The Secretary orders that all condemned carcasses be stamped with the condemnation stamp, and that the inspectors shall cause all such carcasses to be placed in the rendering tanks of the abattoir. I will submit a few facts in relation to some evil conditions that existed at one of the private abattoirs in New York City. And what is true of this one is also true to a greater or less degree of others that came under my observation. From July 1, 1892, to January 1, 1893, there were slaughtered in this abattoir 3601 cows. Out of this number, 492 were diseased, 237 were sick with contagious diseases, and 255 were sick with non-contagious diseases; 15 were at the point of death, as the consequence of their malady, when driven into the shoots to be slaughtered, and three were dressed and hung up that had died on the street en route to the abattoir. Of this number, 492 sick animals, 209 had tuberculosis represented in its varying stages, from the small pearly-like tubercle, as seen on the serous membrane, to complete caseations and calcifications of the tissues involved, and in some instances the carcasses would give forth a very offensive odor. One hundred and thirty-four had pleurisy associated with its complications; 121 had mammitis of the entire udder, and in the majority of cases to the extent of profuse suppurations; eighteen had Texas fever, and had these been left to themselves a few hours longer they would have succumbed. As before stated, three of these died en route to the slaughter-house, and ten had actinomycosis. Out of this total of 492 diseased carcasses, not one of which was fit for human food, the municipal inspector of slaughter-house meats consigned only nine to the offal, that being done in a perfunctory way, and by no means the worse ones were cut down. Were there not a final market for this class of inferior food product in the city of New York, these cows would not have found their way to a slaughter-house. It is within a few years that there has been a demand for flesh of this inferiority. This demand is on the increase. Only recently have the dairymen living within the vicinity of the metropolis been able to find a market for the old, worn-out, thin in flesh, and diseased animals of their herds. But to-day the rural owner of such cattle finds a ready market and fair prices for this grade of neat cattle. It is a common expression among dairymen when considering the question of dispensing with the old cow to hear them say, "I'll ship her to York." In following this product to its final destination we find that, either of necessity or choice, it goes to the occupants of the thickly-packed houses of the foreign-born population. In the face of these facts, and that this demand for unwholesome meats comes from a class of people not Americans by birth or instinct, whose mode of living is such as tends to invite disease, it therefore becomes a matter of more than municipal concern if in the great metropolis of this country conditions are such that it becomes a laboratory for the propagation of contagion and infection, both domestic and foreign. We have not named all the diseases or unwholesome conditions under which the cattle are slaughtered, whose flesh is used as food; but enough has been stated to illustrate the character of existing evils of this nature which naturally confront us with the questions, How can these evils be abated? and, Who are the proper persons to exercise so important a function?

In the first place, we believe this can be best accomplished by the State. Our reasons are obvious. From April to July 1, 1893, we had occasion to visit daily two abattoirs on Johnson Avenue in the city of Brooklyn. These were private slaughter-houses. Both did contract work for other individual dealers. One of these slaughtered anything that was offered; the other dressed only first and second-grade meats. Through the Board of Health the city had meat inspectors also stationed at these abattoirs, whose duty it was to inspect all slaughtered meats for city consumption. Scarcely ever were they on duty, and when on duty it was very seldom they would cut down a diseased carcass. In moral, there was no inspection by the municipal officer at these abattoirs.

There are other reasons why the State should control meat inspection. It would be a recognized central power from which all regulations would emanate; uniform in character, progressive in its tendencies, and accepted as authoritative it would remove it from the bane of politics by passing the civil service, thus placing it upon a plane of moral and intellectual fitness. To do this, and to do it efficiently, we would need such laws as will recognize and meet the emergencies and conditions that are shown to exist. The more elaborate such law, the more beneficent would its results be.

The proper place to carry on meat inspection is in the slaughter-house, and at present it is here that the most unwholesome and unsanitary conditions exist. Men who are sanitarians in ability and adaptation sufficient to meet the demands of their position and its relation to the public, cannot in the nature of things be expected to perform a public duty ankle-deep in filth and blood.

(Be it understood that these conditions relate wholly to private slaughter-houses, and in some instances to the public abattoir.)

Those whose privilege it has been to visit the city abattoirs or those in the country districts must have been impressed that there was need of more sanitary regulations than were observed under its present management. In cities there are many reasons why the private abattoir should be done away with, and every reason why there should be none but the public abattoir. These restrictions would not deprive a single slaughter-man of his business, as each could have his space and time allotted. That there should be centralized abattoirs constructed upon sanitary principles is quite as necessary as that meat should be inspected. A building constructed large enough to take care of the business which would naturally come to it, with ample room for dressing beds, cooling trucks, and storage, with a skylight roof over all the working floors, thoroughly ventilated, the plumbing to be of the most modern and sanitary design, properly underdrained and a one-story building, are the crying needs of the hour along this line of reform.

The question now naturally arises, Who is the proper person to be the inspector of meats? There can be but one answer to this question. It is the veterinarian. He, by profession and practice, becomes the person best fitted to judge of the wholesomeness or unwholesomeness of animal flesh as food. His knowledge of the diseases of the lower animals pre-eminently fits him above all others to discharge so important a public duty.

In conclusion, we are not unmindful of the importance of the subject which our worthy Secretary allotted me to discuss on this occasion. Its relation to the public health is one of vital concern, demanding on all occasions hearty support and aggressiveness in its behalf on the part of the profession. It becomes by its uniqueness the veterinarians' duty in our Commonwealth, and while a duty it becomes a greater pleasure by putting ourselves in line to be numbered among the public benefactors of our time

